# Biomechanical comparison of various implant inclinations and abutment types in a bendable implant system

**DOI:** 10.1186/s12903-025-06610-1

**Published:** 2025-07-19

**Authors:** Esra Bilgi-Ozyetim, Gökçen Dinçer, Ammar Sulaiman, Süleyman Çağatay Dayan, Sevcan Kurtulmus-Yilmaz, Onur Geçkili

**Affiliations:** 1 Department of Prosthodontics, Faculty of Dentistry, Istanbul Yeni Yuzyil University, Istanbul, Türkiye; 2 Department of Prosthodontics, Faculty of Dentistry, Istanbul University, Istanbul, Türkiye; 3 Private Clinic, Istanbul, Türkiye; 4 Program of Dental Technicians, Istanbul University-Cerrahpaşa, Istanbul, Türkiye; 5 Department of Prosthodontics, Faculty of Dentistry, Near East University, Nicosia, Mersin10, Türkiye; 6 Department of Prosthodontics, Dentistry Faculty, Istanbul University, Türkiye. Süleymaniye, Prof. Dr. Cavit Orhan Tütengil Sk. No:4, Istanbul, Fatih, 34116 Türkiye

**Keywords:** Bendable implant, One-piece implant, Finite element analysis, Stress distribution, All-on-4, Angulated implant

## Abstract

**Background:**

This study aims to evaluate the stress distribution in peri-implant bone, implant surfaces, and within the framework material under vertical and oblique loads, considering various angulations and abutment types of bendable one-piece implants in the All-on-4 concept.

**Methods:**

A three-dimensional model of an edentulous mandible was constructed. Six different configurations were modeled according to prosthesis connection type and inclination of the posterior implants based on the All-on-4 protocol. Four one-piece implants were placed in each model and a cobalt-chromium frameworks with 14 mm cantilever lengths were designed. Under vertical and oblique loading conditions, maximum principal stress and minimum principal stress values were obtained for cortical and trabecular bone and von Mises stress values were calculated for implants and frameworks.

**Results:**

Implant angulation, connection type, and loading conditions affected stress distribution in the peri-implant bone, implants, and frameworks. Higher stress values were observed in posterior implants with increased angulation. Screw-retained models demonstrated greater stress values compared to cement-retained ones under both loading conditions.

**Conclusions:**

For optimal stress distribution in peri-implant bone and frameworks, cement-retained prosthesis may be preferred, especially in cases with increased implant angulation in one-piece bendable systems. Additionally, careful consideration of posterior implant angulation and loading conditions is crucial to minimize the excessive stress and to enhance long-term stability.

**Clinical trial number:**

Not applicable.

## Background

The primary objective of contemporary dentistry is to address the functional, aesthetic, and psychological needs of those who have experienced tooth loss for a variety of reasons [1]. Dental implants have emerged as a crucial component of prosthodontic treatment for patients with partial or complete edentulism. It is essential to comprehend the distribution of stress that arises during the phases of mastication and functioning, as the occlusal forces exerted on the prosthesis are transmitted to the adjacent bone. Excessive stress loading can result in the deterioration of osseointegration and subsequent failure of the implant [2].

Unfavorable implant inclination can lead to biomechanical problems, causing harmful stresses on the implants, resulting in typical failures such as screw loosening, abutment fracture, and porcelain veneer chipping [3, 4]. In order to prevent clinical complications arising from the improper placement of implants in two-piece implant systems, a number of dental implant manufacturers offer pre-angled abutments [5]. In some cases, dental implants may be intentionally tilted due to factors such as reduced bone volume, maxillary sinus pneumatization, or posterior bone resorption in the mandible after posterior tooth extraction, making implant placement challenging [6]. Recent research suggests that posterior dental implant tilting up to 45 degrees can be a viable option to bone grafts and sinus augmentation, allowing for less involvement with the maxillary sinuses [7–9].

The All-on-4 design eliminates the need for complex surgical interventions in patients who are entirely missing teeth and have atrophic jaws, using four implants: two axial anterior and two tilted posteriors. These implants are carefully positioned to pass through the sinus floor and make the most of the patient’s remaining bone [10]. By increasing the anteroposterior spread, the All-on-4 concept reduces the distal cantilever length and improves cortical anchorage by permitting the implantation of longer dental implants that are optimally positioned to provide prosthetic support [11]. These clinical situations might be handled with pre-angled abutments in two-piece implant systems. On the other hand, clinicians may face difficulties when using single-piece dental implant systems in the aforementioned cases especially when mounting the angled abutments into the most distal implants.

Single-piece implants offer several advantages, including the elimination of gaps between the implant body and the abutment, preventing loosening of abutments or prosthetic screws, which are common in two-piece systems [12, 13]. Additionally, they prevent epithelial damage and bone resorption while saving time by eliminating the need to remove or adjust gingival formers and abutments during prosthetic procedures [14]. The macro design of the implant neck also reduces the risk of peri-implantitis [15], and the surgical and prosthetic process is faster compared to two-piece systems [16, 17].

In single-piece implants requiring angulation, only solution can be bending the abutments if the system allows the clinicians. However, to the best of authors’ knowledge, no biomechanical studies have yet evaluated the stress distribution in screw-retained and cement-retained restorations using bendable single-piece implant systems. This study aims to use finite element analysis (FEM) to assess stress distribution in screw-retained and cement-retained bendable single-piece implant systems subjected to vertical and oblique forces, utilizing four implant-supported fixed prosthesis. The null hypotheses of this study were that the connection type and the angulation of the posterior implant would have no effect on the von Mises stress distribution in implants, peri-implant bone, or the framework.

## Methods

### Model construction and ethical approval

A three-dimensional (3D) model of a completely edentulous mandible was developed using high-resolution computed tomography (CT) data obtained from the publicly available Visible Human Project, which provides anatomically detailed datasets suitable for biomechanical simulations. The study protocol was approved by the Istanbul Yeni Yuzyil University Science and Health Sciences Research Ethics Committee (No. 2022/05-860).

### Mandibular geometry acquisition and processing

CT images were reconstructed with a slice thickness of 0.1 mm and imported into 3D Slicer (Kitware, Clifton Park, NY, USA) in Digital Imaging and Communications in Medicine (DICOM) format. This open-source software was employed for image segmentation and 3D model generation. Semi-automatic segmentation was performed using appropriate Hounsfield Unit (HU) thresholds to isolate the mandibular bone, followed by manual refinement and region-growing techniques to ensure anatomical accuracy, especially near cortical-trabecular boundaries and critical landmarks such as the mental foramen and mandibular canal.

The 3D surface model was generated using the marching cubes algorithm and smoothed with Laplacian filters to eliminate voxel artifacts while preserving essential morphological features related to stress transmission.

Further refinement was conducted in ANSYS SpaceClaim (Ansys Inc., Canonsburg, PA, USA). A 2-mm cortical bone shell was created by offsetting the outer surface of the mandible, and the trabecular bone was modeled by referencing the internal geometry. Mucosal thickness was defined based on the gingival height of the abutments. Implants were placed unicortically, with all regions except the neck embedded in the trabecular bone. All components were spatially aligned and positioned in the appropriate coordinates in 3D space within ANSYS SpaceClaim, ensuring an anatomically faithful representation for subsequent biomechanical analyses.

### Prosthetic modeling and experimental design

The mandible and prosthesis models were combined with computational models of the implants and prosthetic components to represent an implant-supported mandibular fixed complete prosthesis. Six different test models based on the All-on-4 protocol (Table 1) were created according to the retention type of the prosthesis and the inclination of the posterior implants. Four one-piece implants, based on the dimensions of Mode Provo implants (Mode Implant / Mode Medikal San. Tic. Ltd. Şti., İstanbul, Türkiye), were placed in each model, and 3D computer-aided design (CAD) models were obtained from the manufacturer.

**Table 1:** Study models with different posterior implant inclinations and prosthesis retention types

Model	Implant location and inclination	Retention type
Model 1	Anterior: Lateral incisor region, parallel Posterior: 2nd premolar region, 17° distal inclination	Screw-retained
Model 2	Anterior: Lateral incisor region, parallel Posterior: 2nd premolar region, 17° distal inclination	Cement-retained
Model 3	Anterior: Lateral incisor region, parallel Posterior: 2nd premolar region, 30° distal inclination	Screw-retained
Model 4	Anterior: Lateral incisor region, parallel Posterior: 2nd premolar region, 30° distal inclination	Cement-retained
Model 5	Anterior: Lateral incisor region, parallel Posterior: 2nd premolar region, 45° distal inclination	Screw-retained
Model 6	Anterior: Lateral incisor region, parallel Posterior: 2nd premolar region, 45° distal inclination	Cement-retained

**Fig. 1:**
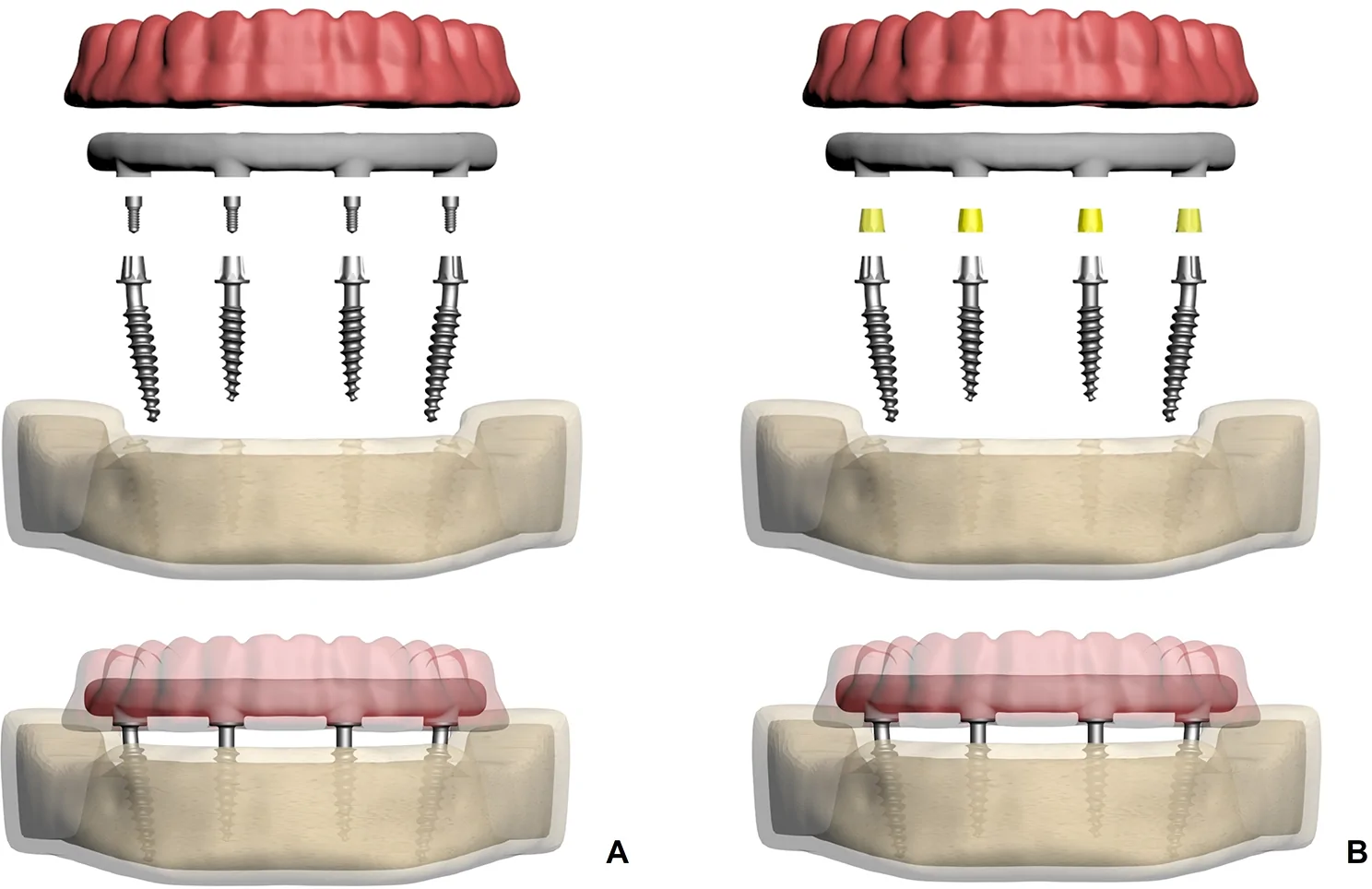
(**A**) Representation of the screw-retained bendable one-piece implant model, including the implant, prosthetic framework, and screw-retention components. (**B**) Representation of the cement-retained bendable one-piece implant model, illustrating the implant, prosthetic framework, and cement layer used for retention

**Fig. 2:**
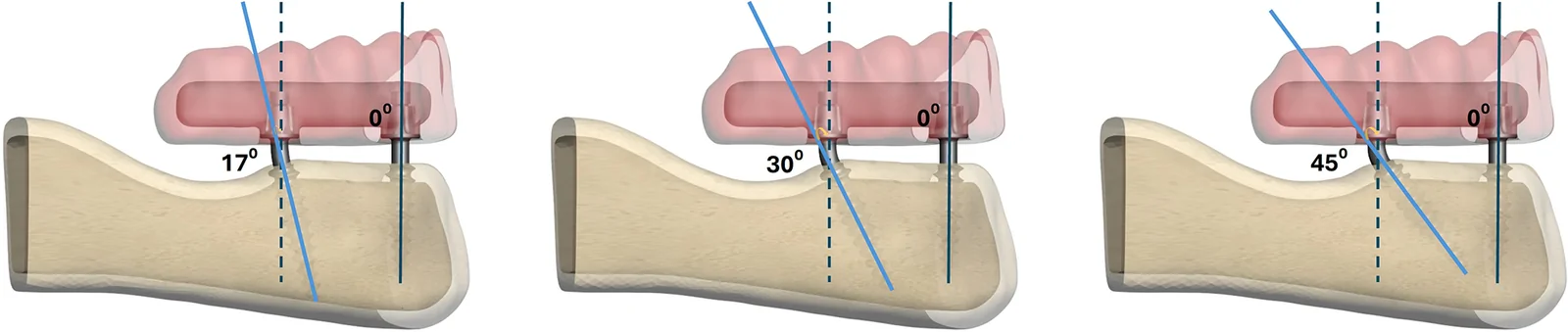
The inclination of posterior implants varies across models as 17°, 30°, and 45°

In the screw-retained test groups (models 1, 3, and 5), data from the Mode Provo-S implants were used (Fig. 1a), while in the cement-retained test groups (models 2, 4, and 6), data from the Mode Provo-C implants were modeled (Fig. 1b). In all models, the two anterior implants (3.5 mm in diameter and 12 mm in length) were positioned vertically at the lateral incisor sites. The posterior one-piece implants (3.5 mm in diameter and 15 mm in length) were modeled as bent at the neck, with inclinations of 17° (models 1 and 2), 30° (models 3 and 4), 45° (models 5 and 6) and placed in the second premolar regions (Fig. 2).

In cement-retained groups, a 20-µm polycarboxylate cement layer was modeled at the superior region of the implant, conforming to the abutment surface. Cobalt-chromium frameworks incorporating a 14-mm distal cantilever were designed for all models. All parts were meshed and assembled in ANSYS SpaceClaim, and mesh compatibility was ensured in ANSYS Workbench (Table 2).

**Table 2:** Quantitative model information

Materials	Elastic Modulus (MPa)	Poisson Coefficient (ν)
Cortical Bone	13,700	0.3
Trabecular Bone	1370	0.3
Feldspathic Porcelain	82,800	0.35
Cobalt-Chrome (CoCr)	218,000	0.33
Titanium	110,000	0.3
Cement	5110	0.35

### Mesh generation and convergence validation

A volumetric finite element mesh was constructed using 4-node tetrahedral (TET4) elements to accurately represent the complex anatomical geometry of the mandible and implant-supported prosthetic system. Element sizes ranged from 0.1 to 0.5 mm for cortical and trabecular bone, and from 0.1 to 0.25 mm for implants, abutments, and metallic superstructures. Local mesh refinement was applied in regions with high stress gradients, particularly around the implant–abutment interfaces. On average, the meshed models contained approximately 836,596 nodes and 3,479,223 elements.

To ensure solution accuracy without excessive computational demand, a mesh convergence analysis was conducted on a representative submodel. Key outcome measures included the maximum von Mises stress in implants, principal stresses in peri-implant bone, and reaction forces at boundary nodes. The mesh was refined iteratively, and the relative error between successive simulations was calculated using the formula:

$${\rm{Relative}}\,{\rm{Error}}(\% )\, = \,\left( {{\matrix{{\rm{Value(Updated}}\,{\rm{Mesh)}} - \hfill \cr{\rm{Value(Previous}}\,{\rm{Mesh)}} \hfill \cr} \over {{\rm{Value(Previous}}\,{\rm{Mesh)}}}}} \right)\, \times \,100$$

Refinement was continued until the relative error dropped below the predefined threshold of 2–3%, confirming mesh convergence and numerical stability (Tables 3 and 4). Mesh quality was further assessed using geometric criteria such as skewness (> 80°) and minimum edge length (> 0.001 mm); any elements that failed to meet these criteria were corrected accordingly. The final mesh characteristics for each model are summarized in Table 5.

**Table 3:** Implant convergence test results

Mesh Size (mm)	Estimated Stress (MPa)	Relative Error (%)
0.5	68.96	-
0.3	75.06	8.84
0.2	78.63	4.75
0.1	80.94	2.93

**Table 4:** Bone (around implant) convergence test results

Mesh Size (mm)	Estimated Stress (MPa)	Relative Error (%)
0.5	6.56	-
0.3	7.34	11.89
0.2	7.75	5.57
0.1	7.97	2.80

**Table 5:** Mesh characteristics of the finite element models

	Total # of Nodes	Total # of Elements
Model 1	836,596	3,479,223
Model 2	872,903	3,561,709
Model 3	818,812	3,406,392
Model 4	855,404	3,490,379
Model 5	868,215	3,618,125
Model 6	904,817	3,702,547

### Implant geometry and discretization

Implant geometries were obtained directly from the manufacturer as CAD files to ensure dimensional accuracy. These models were imported into ANSYS Workbench and discretized using the same meshing strategy. Mesh refinement was applied in curved and high-stress areas to accurately capture load distribution behavior.

### Model count and simulation overview

Six finite element models were developed, and two different linear static analyses were performed on each model under distinct loading scenarios, resulting in a total of 12 simulations. Simulations were conducted using LS-DYNA under quasi-static assumptions.

### Loading and boundary conditions

**Fig. 3:**
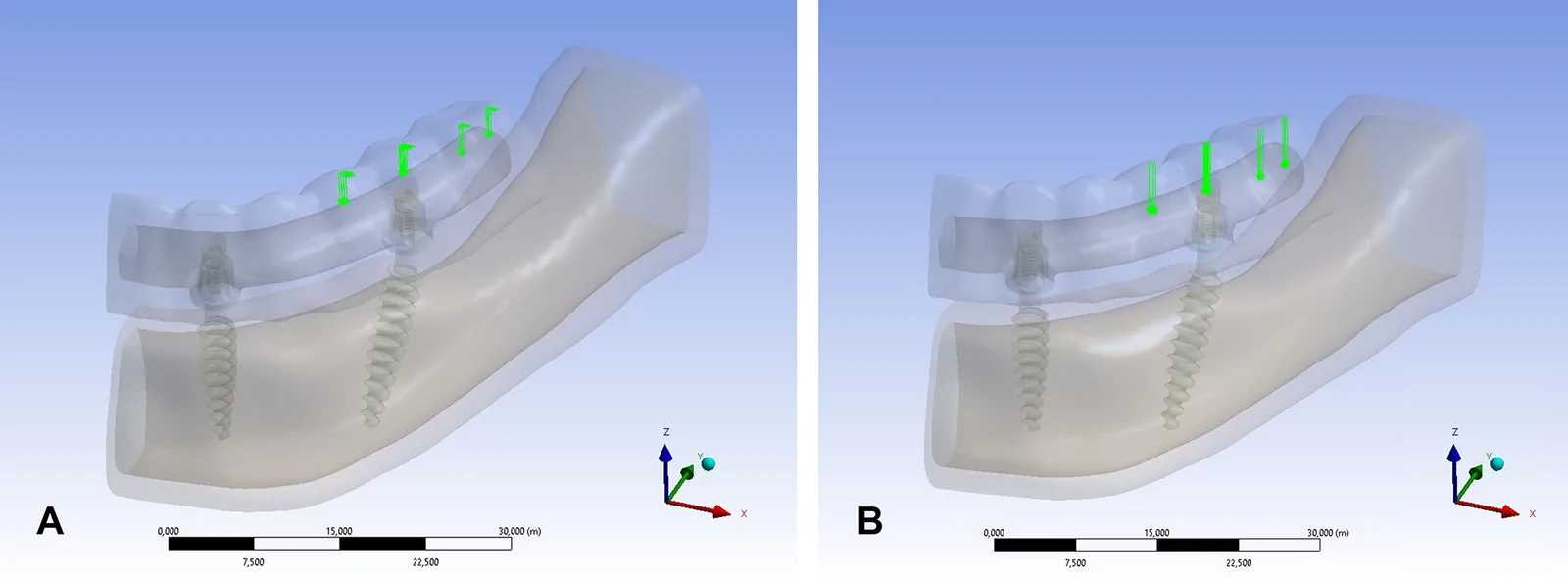
Boundary conditions applied in the finite element analysis. (**A**) Oblique loading scenario, (**B**) Vertical loading scenario

Two loading scenarios were defined to simulate functional masticatory forces:

Oblique loading: A 150 N force at a 75° angle in the linguo-buccal direction was applied to the buccal cusps of the 1st premolar, 2nd premolar, and 1st molar teeth (Fig. 3a).

Vertical loading: A vertical 150 N force was applied to the same regions (Fig. 3b).

To avoid stress singularities, loads were distributed over peripheral nodes within the contact zones. Boundary conditions were assigned to appropriately constrain mandibular movement during load application.

## Results

### Stresses in peri-implant bone

**Fig. 4:**
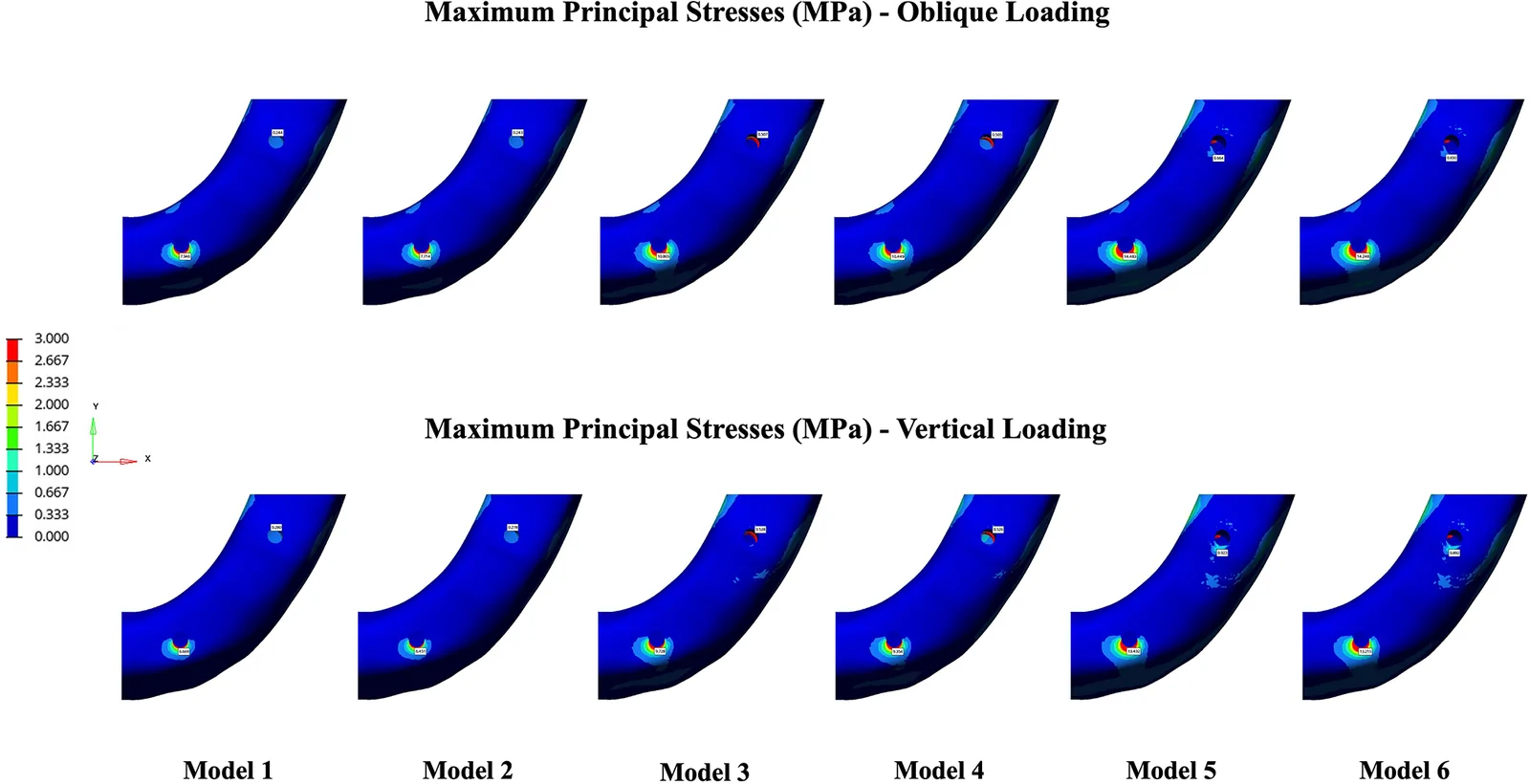
Pmax maps in the cortical bone under oblique (top row) and vertical (bottom row) loading conditions. The images are unprocessed except for merging multiple simulation outputs for layout clarity. No changes to brightness, contrast, or color have been applied. The color scales and data remain as originally generated from the finite element analysis

**Fig. 5:**
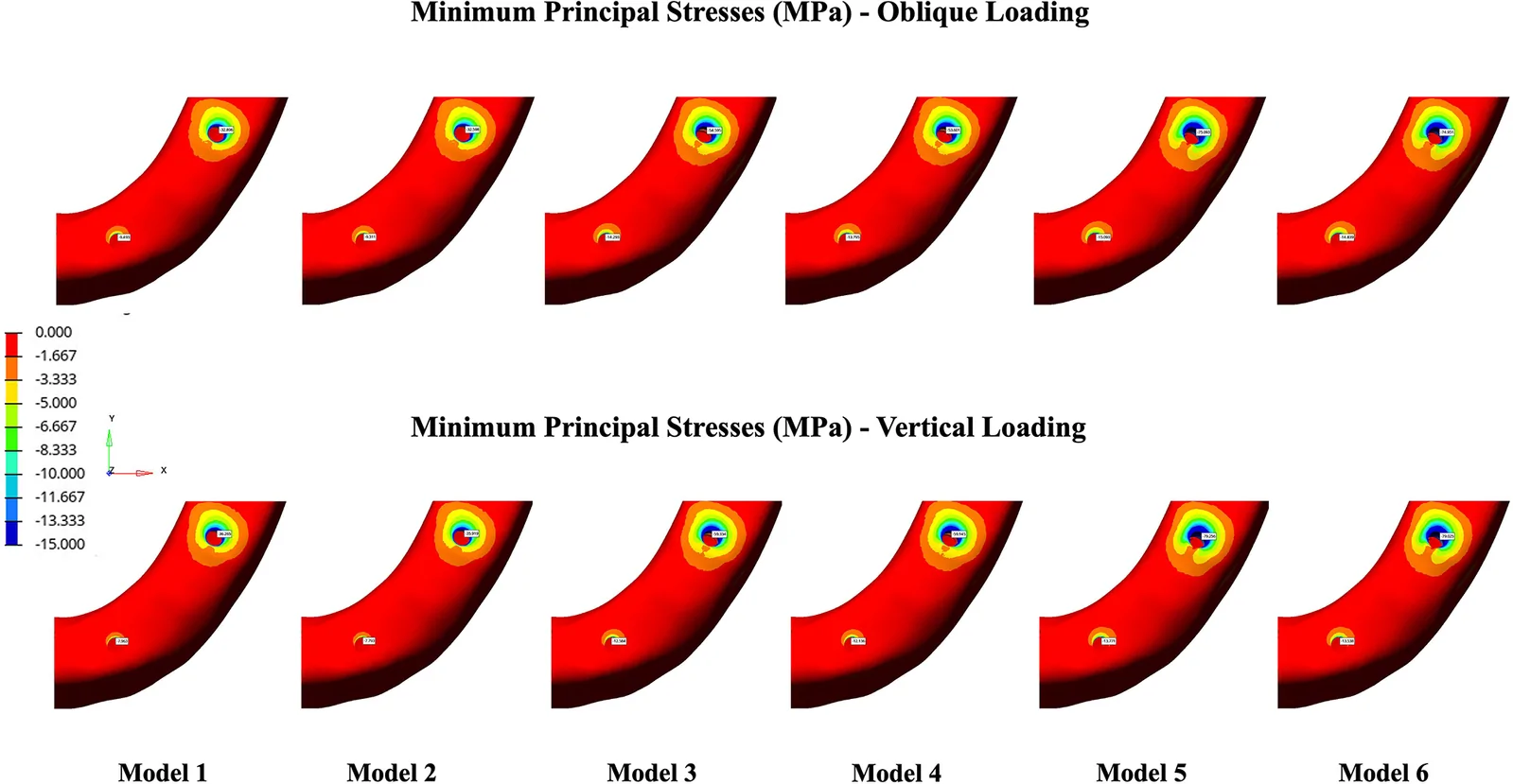
Pmin maps in the cortical bone under oblique (top row) and vertical (bottom row) loading conditions. The images are unprocessed except for merging multiple simulation outputs for layout clarity. No changes to brightness, contrast, or color have been applied. The color scales and data remain as originally generated from the finite element analysis

The Pmax and Pmin stress distributions in the cortical bone are depicted in Figs. 4 and 5. According to the analyses, implant angulation, implant-framework connection type, and loading conditions were found to be effective on the stress distributions in the peri-implant bone. Both Pmax and Pmin stresses in anterior and posterior peri-implant bone increased with the increase in the angulation degree of posterior implant. In the anterior implant region, vertical forces produced slightly lower Pmax and Pmin values in comparison to oblique forces, whereas vertical loading composed higher stress values in the posterior implant region. Stress values were lower in cement-retained models (2, 4, and 6) compared to their screw-retained counterparts (1, 3, and 5).

### Maximum principal stresses

Regardless of the loading conditions, the highest Pmax values were observed in the anterior and posterior implant regions of Model 5 (45° angulation, screw-retained); while the lowest values were observed in both regions of Model 2 (17° angulation, cement-retained). In all models, highest Pmax values were concentrated around the mesial and buccal aspects of the implant necks.

**Fig. 6:**
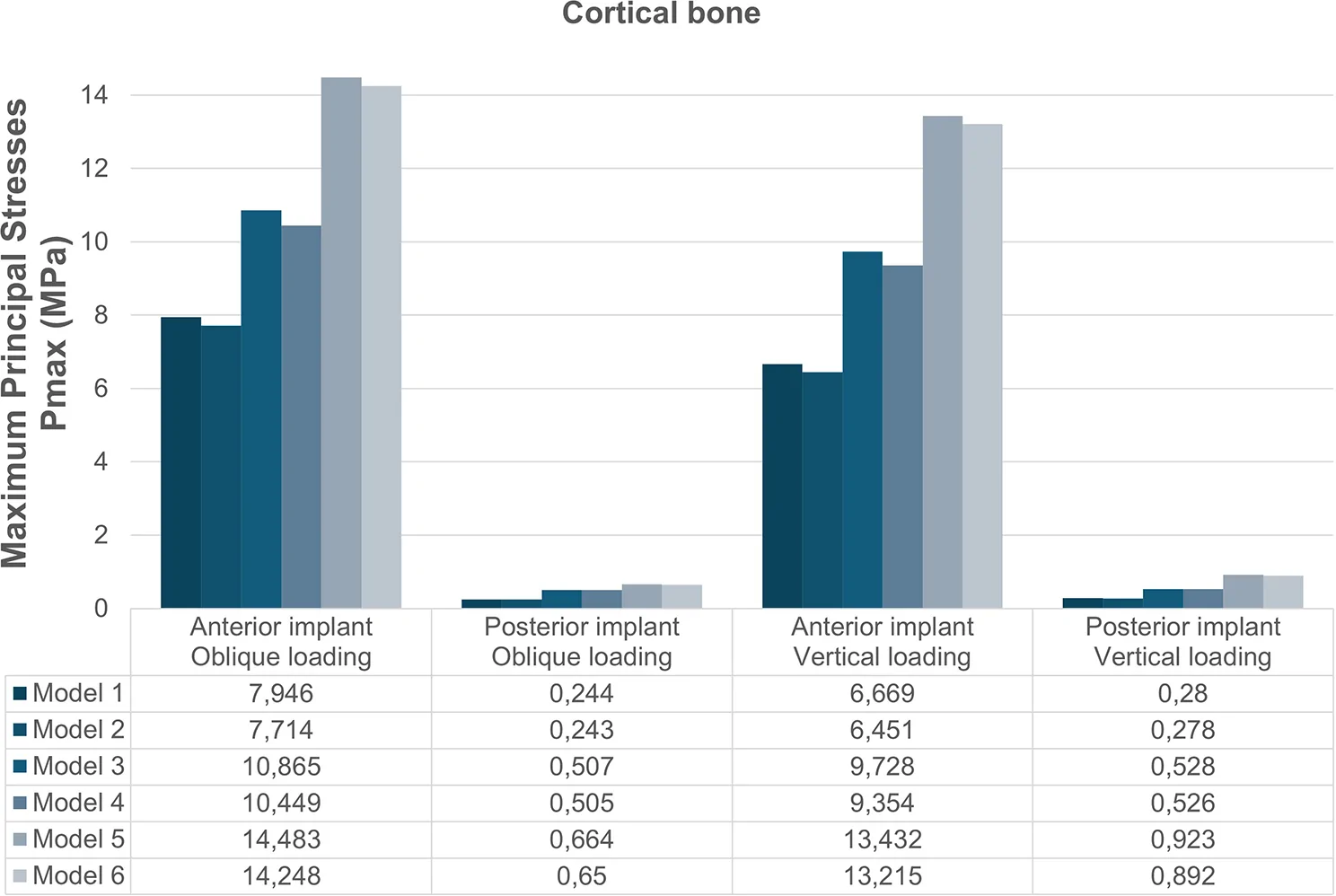
Distribution of Pmax values in the cortical bone under oblique and vertical loading conditions for all models

The highest Pmax values determined under oblique and vertical loadings were in the anterior implant region of Model 5 with the values of 14.483 MPa and 13.432 MPa, respectively. The lowest Pmax values, recorded in the posterior implant region of Model 2 were 0.243 MPa under oblique loading and 0.278 MPa under vertical loading (Fig. 6).

### Minimum principal stresses

The highest Pmin values were identified in posterior implant region of Model 5 whereas the lowest values were found in Model 2, regardless of the loading direction. According to stress maps, the highest compressive stresses were determined on the distal aspects of the cortical bone around the posterior implants.

**Fig. 7:**
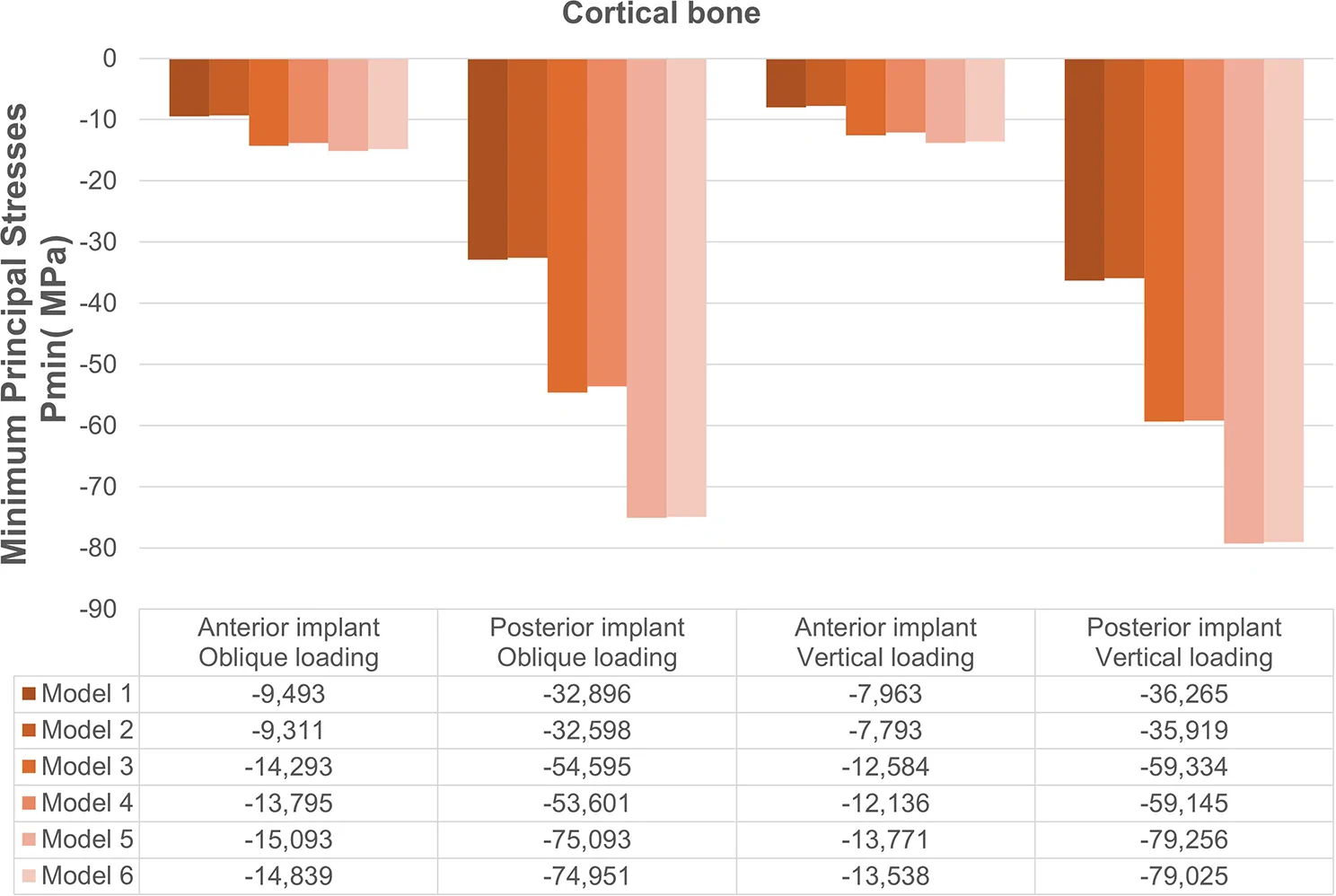
Distribution of Pmin values in the cortical bone under oblique and vertical loading conditions for all models

Among the models analyzed, the posterior implant region of Model 5 demonstrated the highest Pmin stresses, with values of − 75.093 MPa under oblique loading and − 79.256 MPa under vertical loading. The lowest Pmin stresses were identified in Model 2 as − 32.598 MPa and − 35.919 MPa during oblique and vertical loadings, respectively (Fig. 7).

### Stresses in implants

**Fig. 8:**
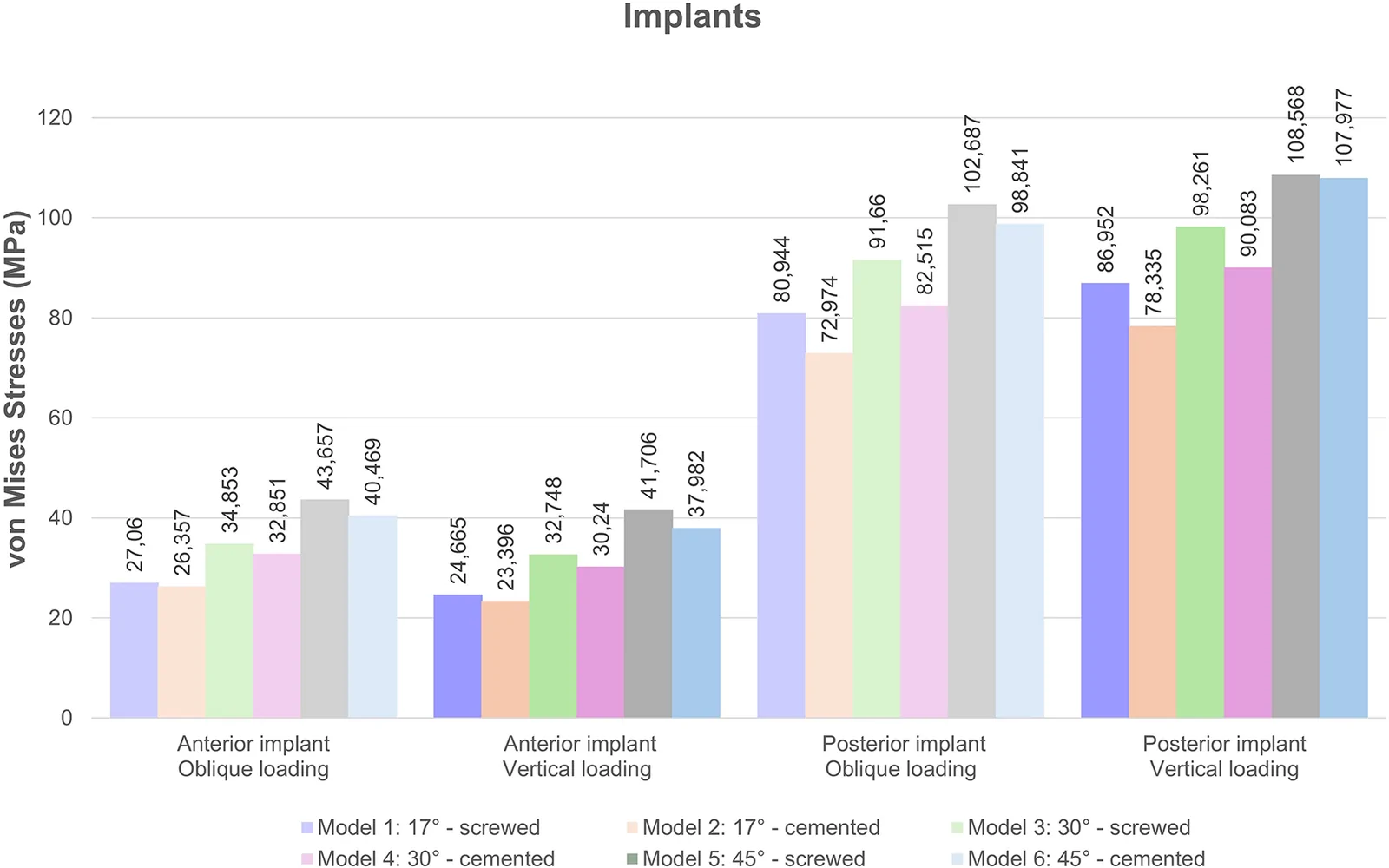
Comparison of von Mises stress values in the implants under oblique and vertical loading

**Fig. 9:**
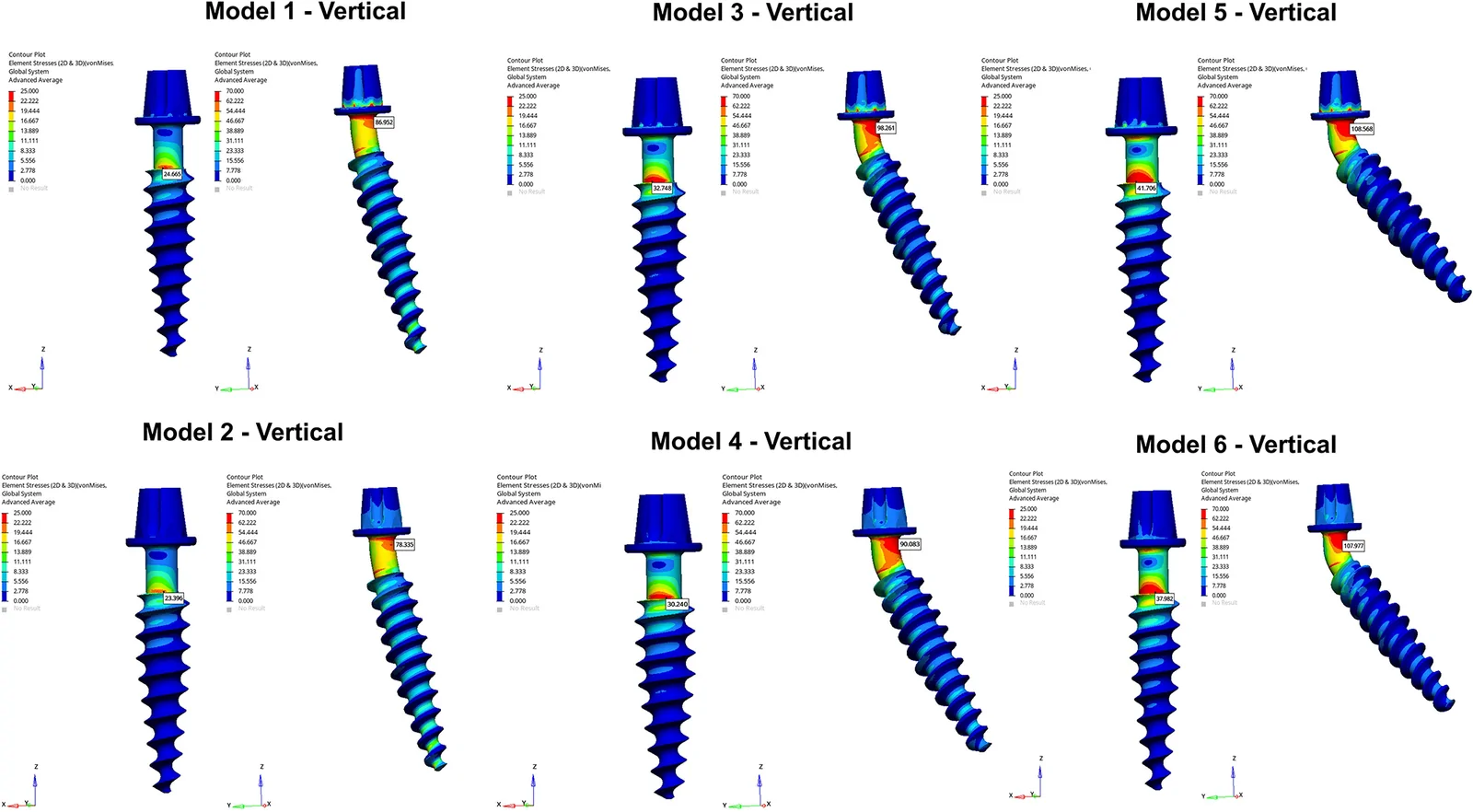
Color-coded stress maps indicating von Mises stress distributions in the implant structures under vertical loading. The images are unprocessed except for merging multiple simulation outputs for layout clarity. No changes to brightness, contrast, or color have been applied. The color scales and data remain as originally generated from the finite element analysis

**Fig. 10:**
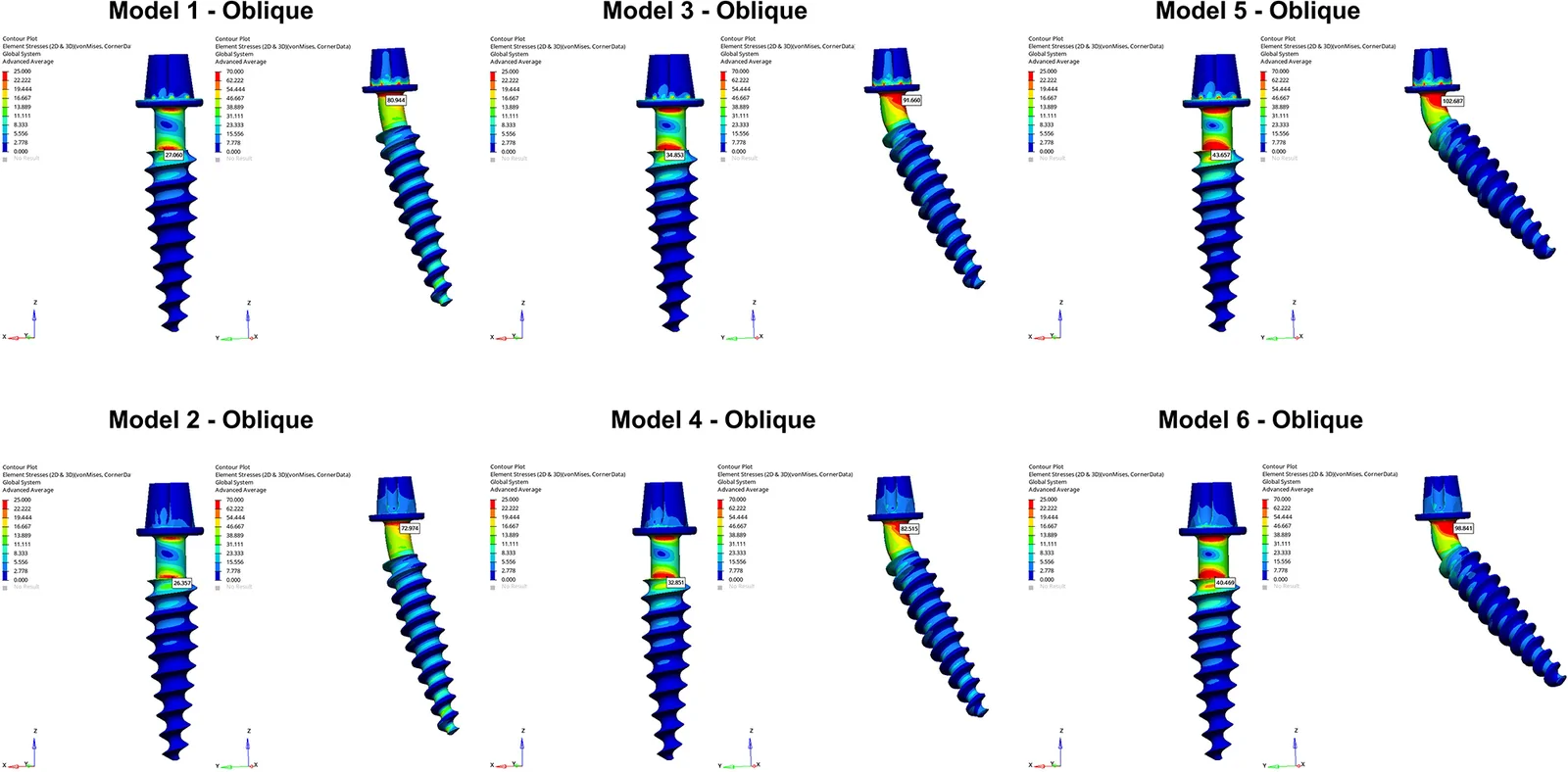
Color-coded stress maps indicating von Mises stress distributions in the implant structures under oblique loading. The images are unprocessed except for merging multiple simulation outputs for layout clarity. No changes to brightness, contrast, or color have been applied. The color scales and data remain as originally generated from the finite element analysis

The angulation of the posterior implants and the connection type influenced the von Mises stresses detected on the implants. Regardless of the loading conditions, higher stress values were determined in posterior implants in comparison to anterior implants. Von Mises stresses increased with the increase of the implant angulation (Fig. 8). Screw-retained models exhibited higher stresses than cemented-connection models in both anterior and posterior implants. Color-coded stress maps revealed that von Mises stresses were concentrated in the neck regions of anterior and posterior implants. In posterior implants, highest stresses were observed in the mesial aspect of the implant neck closer to the abutment while in anterior implants stresses were higher in buccal aspect of the implant neck near the crestal region (Figs. 9 and 10).

In anterior implants, oblique forces generated higher stresses while in posterior implants higher stress values were observed under vertical forces. Among the models, for both anterior and posterior implants, the highest Von Mises stress values were determined in Model 5 under both loading conditions. For anterior implants, the highest stress value was recorded as 43.657 MPa under oblique loading and for posterior implants the highest value was detected as 108.568 MPa under vertical loading in Model 5. The lowest von Mises stresses were identified in anterior and posterior implants of Model 2 with the values of 23.396 MPa and 72.794 MPa, respectively (Fig. 8).

### Stresses in framework

**Fig. 11:**
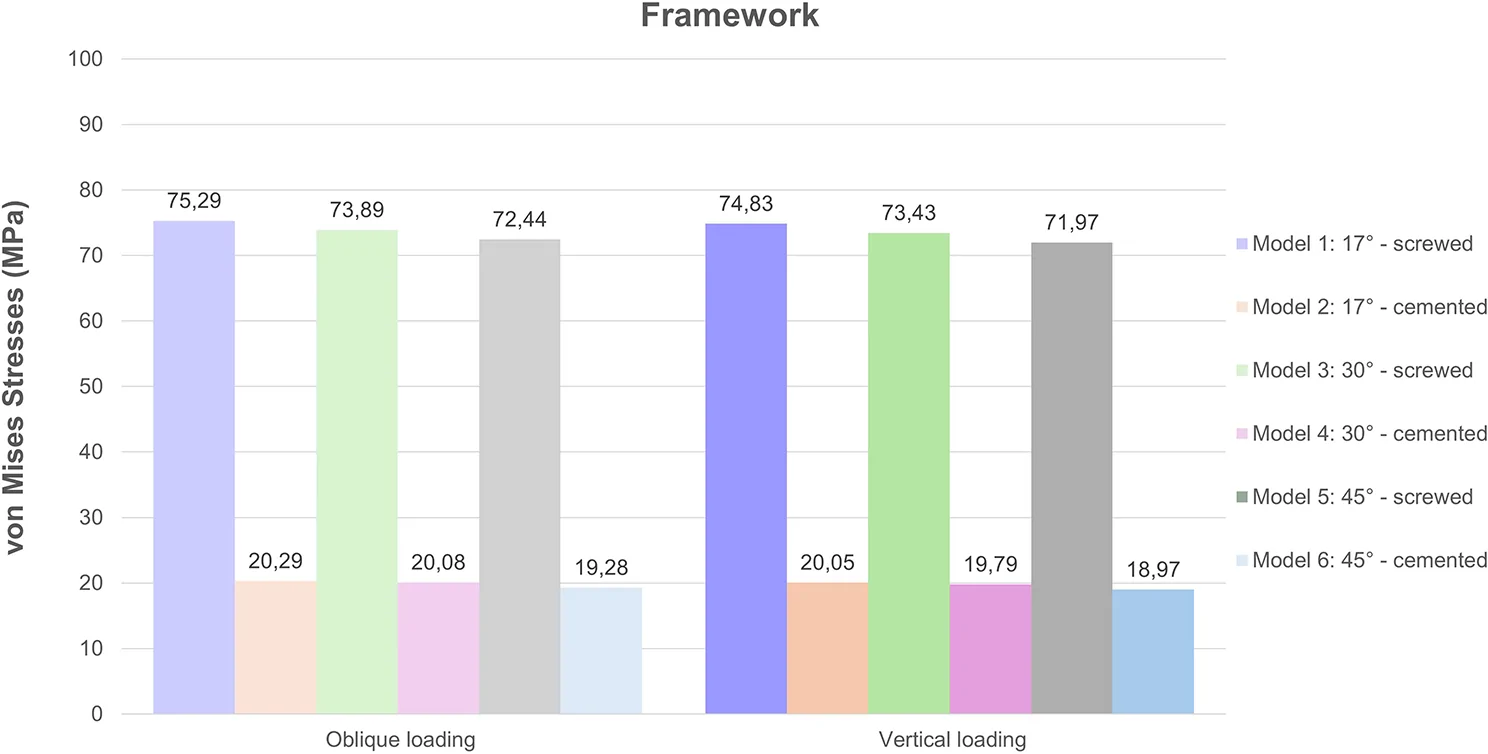
Distribution of von Mises stress values in the prosthetic framework under oblique and vertical loading

Connection type significantly affected the stress values determined in frameworks. Under both oblique and vertical loading conditions, the von Mises stresses observed in the frameworks of screw-retained models were more than 3.5 times higher than those in cement-retained models. The increase in the angulation of posterior implants resulted in a slight decrease in the stress values determined in the framework. The highest stresses were found in Model 1 (17° angulation, screw-retained) with values of 75.297 MPa and 74.827 MPa, under oblique and vertical forces, respectively (Fig. 11). Color maps indicated that stress was concentrated around the posterior implant regions in all models. More uniform stress distribution was observed in cement-retained models.

## Discussion

This study evaluated the effects of different implant inclinations and implant-abutment connections on the biomechanical behavior of bendable single-piece implants and stress distribution across the peri-implant bone and prosthetic framework. The null hypotheses of the study was rejected since the findings of the finite element analysis revealed that both connection type and the implant angulation had significant effects on stress distribution.

All-on-4 treatment concept has been established as a reliable option in the treatment of edentulous arches with fixed restorations according to the long-term clinical outcomes [18–21]. Angulated implants allow the placement of longer implants in the posterior region, which increases the contact area between bone and implant and also improves implant stability [22]. Furthermore, tilting the posterior implant may eliminate the necessity for grafting procedures and reduce the cantilever length [23].

To fabricate full arch prosthetic restorations, the angulation of the tilted implants can be adjusted using various solutions, such as angled multi-unit abutments, angulated screw channels, or built-in angle corrected implants [3, 22]. These approaches endeavor to optimize prosthetic design while addressing the clinical limitations. However, it has been reported that the use of angulated abutments might result in technical complications, especially screw loosening [3]. In single-piece bendable Provo implants, the correction of the implant angulation is achieved without the need for additional components reducing the risk of potential mechanical failures. Single-piece implants have advantages over two-piece implant systems including minimizing micromotion and consequently reducing inflammatory responses in the surrounding tissues [24]. Furthermore, finite element analyses have reported that single-piece implants exhibit lower stress concentrations [25, 26] and allow for more uniform stress distribution along the implant body in All-on-4 concept [27] compared to two-piece implants. However, studies on single-piece bendable remain limited. Therefore, the current study aimed to investigate the stress distribution in implants, peri-implant bone, and framework when single-piece bendable implants are connected to the framework using either screw or cement.

In the present study, the cantilever length and implant lengths were standardized in all models, regardless of the angulation of the posterior implants to eliminate variables related to the lever arm effect and ensure that the detected differences in stress distributions could be attributed only to the changes in implant angulation and the bending of implant neck. The results of the present study revealed that angulation of the posterior implant significantly affected the stress distribution in peri-implant bone. Increased angulation resulted in higher Pmax and Pmin values indicating that higher implant inclinations compose greater stress concentration in cortical bone that is consistent with previous studies [28, 29] in which cantilever lengths were standardized among the groups. This finding may be attributed to increased bending moments in relation to higher implant angulations since the forces distributed unevenly across the implant and led to localized stress concentrations in the cortical bone.

In cases of total edentulism with multiple implants, screw-retained restorations are usually indicated due to their predictable retrievability, which facilitates hygiene maintenance and possible repairs [30]. Additionally, the elimination of excess cement, which has been reported to be a risk factor for biological complications such as peri-implant diseases, is achieved with screw-retained restorations [31]. On the other hand, cement-retained restorations can achieve a passive fit through the cement layer gap especially when the implants are not placed ideally [32]. Screw-retained and cement-retained restorations exhibit different biomechanical behaviors in full-arch implant supported prosthesis which may affect the stress distribution across the implant, peri-implant bone, and prosthetic components. The results of this study showed that von Mises stresses in screw-retained frameworks were consistently higher than those in cement-retained frameworks under both loading conditions. In vertical loading, screw-retained models exhibited stress values ranging between 71.97 MPa and 74.83 MPa, whereas cement-retained models displayed significantly lower stress values, ranging between 18.97 MPa and 20.05 MPa. Similarly, under oblique loading, von Mises stresses in screw-retained frameworks were between 72.44 MPa and 75.29 MPa, compared to 19.28 MPa to 20.29 MPa in cement-retained frameworks. In screw-retained restorations, the rigid connection between the implant and prosthetic framework often leads to localized stress concentrations, particularly at the implant-abutment interface and peri-implant cortical bone, which may increase the risk of mechanical complications such as screw loosening or implant fatigue. In contrast, cement-retained restorations introduce a degree of elasticity at the connection interface, facilitating a more uniform distribution of occlusal forces. This reduces stress peaks in the peri-implant bone, particularly in the cortical region, potentially lowering the risk of bone resorption [33]. The results of this study are consistent with previous research, suggesting that screw-retained restorations revealed higher stress concentrations in the implants and framework [33–35].

Excessive occlusal loads applied to dental implants can negatively affect the long-term success of implant-supported restorations [36]. When applying FEM to dental implants, it is important to consider not only axial loads and horizontal forces (moment-causing loads) but also a combined load (oblique occlusal force) because the latter represents more realistic occlusal directions and, for a given force, will cause the highest localized stress in cortical bone [37]. The enduring success of implant-supported prostheses heavily hinges on the occlusal loads they are subjected [36, 38].

To simulate average posterior bite forces, previous studies applied oblique and axial loads of 100–150 N on dental implants, particularly those inclined at 30–45 degrees [39–43]. In accordance with these studies, in this study, vertical and oblique (linguo-buccal direction) forces of 150 N were applied to the 1st and 2nd premolars and the 1st molar. Previous research [44–47] has demonstrated that oblique loads induce higher stress concentrations on implants, peri-implant bone, and prosthetic components, significantly altering stress distribution patterns. Consistent with these findings, the present study revealed that oblique loading resulted in higher von Mises stress values in the prosthetic framework and in anterior implants placed vertically. Additionally, Pmax and Pmin values in the cortical and trabecular bone of the anterior region were higher under oblique loading than under vertical loading.

Akça & Iplikcioglu [48] reported that exceeding the von Mises stress value of 550 MPa, which corresponds to the yield strength of a titanium implant, could potentially lead to implant failure. In the current study, von Mises stress values ​​over 550 MPa were not detected on the substructure material and implants under oblique and vertical loads. The highest value obtained in the study was 108.568 MPa observed in posterior implants placed at a 45-degree angle under vertical load.

The reliability of the findings was enhanced through careful standardization during the finite element modeling process. Anatomically accurate mandibular geometry was obtained from CT data, and segmentation of cortical and trabecular bone was performed using appropriate Hounsfield unit thresholds. All materials were defined as homogeneous, isotropic, and linearly elastic. Each model was constructed with a fine tetrahedral mesh exceeding 3 million elements, and bonded contacts were used to simulate fixed interfaces without relative motion. Implant dimensions and cantilever lengths were standardized across all models, allowing stress differences to be attributed solely to implant angulation and connection type. These modeling decisions improve the validity of the observed outcomes, particularly in the comparison of cement- and screw-retained frameworks under increased posterior angulation.

This study has several limitations. First, although FEM provides valuable insights into biomechanical behavior, it cannot fully replicate the complex nature of masticatory forces, which vary in magnitude, direction, and duration in vivo. Moreover, the cantilever length was standardized at 14 mm across all models, which, while allowing for controlled comparisons, may not fully represent the varying clinical scenarios seen in full-arch rehabilitations. Only a single prosthetic material was evaluated in this study, whereas different materials could influence stress distribution and overall biomechanical performance. Further studies evaluating different prosthetic materials, dynamic loading conditions, and long-term clinical outcomes are needed.

## Conclusions

Within the limitations the following conclusions can be drawn:

1. Increasing the angulation of posterior implants results in higher stress values in the cortical and trabecular bone, as well as in anterior and posterior implants, but these values are in the safe range and far below the yield strength of the implants which may cause failure.
2. Cement-retained frameworks exhibit significantly lower stress values compared to screw-retained frameworks under both oblique and vertical loads.
3. Higher stress values occur in posterior implants compared to anterior implants, particularly under vertical loading, whereas anterior implants experience greater stress under oblique forces.

## Data Availability

The datasets used and/or analyzed during the current study are available from the corresponding author upon reasonable request.

## Abbreviations

FEM: Finite element method
3D: Three-dimensional
CT: Computed tomography
DICOM: Digital imaging and communications in medicine
CoCr: Cobalt-Chrome
CAD: Computer-aided design
HU: Hounsfield Unit
