# Correction: Biomechanical comparison of various implant inclinations and abutment types in a bendable implant system

**DOI:** 10.1186/s12903-026-10416-0

**Published:** 2026-11-09

**Authors:** Esra Bilgi-Ozyetim, Gökçen Dinçer, Ammar Sulaiman, Süleyman Çağatay Dayan, Sevcan Kurtulmus-Yilmaz, Onur Geçkili

**Affiliations:** 1 Department of Prosthodontics, Faculty of Dentistry, Istanbul Yeni Yuzyil University, Istanbul, Türkiye; 2 Department of Prosthodontics, Faculty of Dentistry, Istanbul University, Istanbul, Türkiye; 3 Private Clinic, Istanbul, Türkiye; 4 Program of Dental Technicians, Istanbul University-Cerrahpaşa, Istanbul, Türkiye; 5 Department of Prosthodontics, Faculty of Dentistry, Near East University, Nicosia, Mersin10 Türkiye; 6 Department of Prosthodontics, Dentistry Faculty, Istanbul University, Süleymaniye, Prof. Dr. Cavit Orhan Tütengil Sk. No:4, Fatih, Istanbul, 34116 Türkiye

**Correction: BMC Oral Health 25, 1213 (2025)**

**https://doi.org/10.1186/s12903-025-06610-1**

In this article [1] the ethics committee approval number was written incorrectly in the “Materials and Methods” and “Declarations – Ethical approval” sections.

The currently published statement is: “The study protocol was approved by the Istanbul Yeni Yuzyil University Science and Health Sciences Research Ethics Committee (No. 2022/05-860).”

The correct statement should be: “The study protocol was approved by the Istanbul Yeni Yuzyil University Science and Health Sciences Research Ethics Committee (No. 2023/05-1063).”
